# Identification of Genes Essential for Antibiotic-Induced Up-Regulation of Plasmid-Transfer-Genes in Cephalosporin Resistant *Escherichia coli*

**DOI:** 10.3389/fmicb.2019.02203

**Published:** 2019-09-24

**Authors:** Gang Liu, John Elmerdahl Olsen, Line Elnif Thomsen

**Affiliations:** Department of Veterinary and Animal Sciences, Faculty of Health and Medical Sciences, University of Copenhagen, Copenhagen, Denmark

**Keywords:** *Escherichia coli*, antibiotic induced conjugation, cefotaxime, *bla*_CTX–M–1_ resistance plasmid, transfer genes

## Abstract

Bacterial conjugation is one of the most important mechanisms for spread of antibiotic resistance among bacteria. We have previously demonstrated that cefotaxime (CTX) exposure up-regulates expression of Type-IV conjugation transfer genes, and that this leads to increased transfer of a *bla*_CTX–M–__1_ encoding IncI1 resistance plasmid pTF2 in *Escherichia coli*. To elucidate the underlying mechanisms, a search for genes that are essential for the up-regulated expression of the transfer (*tra*) genes in the presence of CTX was undertaken. We constructed a reporter gene-fusion strain MG1655/pTF2 Δ*traF*:*lacZ* where the promoter region of the *traF*-gene of the plasmid pTF2 was fused with a *lacZ* on the native plasmid. Random mutagenesis mediated by Tn5 transposon was carried out in the strain, and seven genes (*rfaH*, *yhiN*, *waaP*, *waaQ*, *gnd*, *pgl*, and IS*Ecp1*) were identified where insertion prevented CTX-induced up regulation of *traF*. Site-specific mutagenesis was carried out, and for all seven mutants, gene deletions abolished the CTX induced up-regulation of *traF*, and the increased conjugation transfer of the plasmid in the presence of CTX was no longer observed. In addition, the deletion of the genes also abolished CTX induced expression of the *bla*_CTX–M–__1_ gene. Our results suggested that through CTX induced induction of the identified genes, *bla*_CTX–M–__1_ expression increased, which led to up-regulation of *traF* and plasmid transfer. These data reveal that a number of chromosomally encoded genes contribute to the antibiotic induced up-regulation of the conjugation machinery of plasmids, and such genes may be future targets to prevent antibiotic induced spread of resistance plasmids.

## Introduction

Conjugation allows bacteria to transfer genetic material from one cell to another via cell to cell contact (Llosa et al., 2002). It has been recognized as one of most important contributors for dissemination of antimicrobial resistance genes (Bennett, 2008). Conjugative plasmids-transfer in Gram-negative bacteria requires the expression of transfer (*tra*) genes involved in DNA transfer and replication, and in mating pair formation (De La Cruz et al., 2010). *tra* genes encode relaxases which are required for processing the DNA and accessory proteins, which are able to recognize the origin of transfer (*oriT*) and to cut the DNA molecule at the *nic* site (Koraimann and Wagner, 2014). Plasmid-conjugation is primarily mediated by Type IV secretion systems (T4SSs), which are multi-protein complexes located in the membrane of the cell and which are able to support the donor and recipient mating-pair (Frost and Koraimann, 2010).

A substantial amount of data suggests that sub-inhibitory concentrations of antibiotics may significantly increase the conjugation transfer frequency both *in vitro* and in the animal gut (Barr et al., 1986; Stevens et al., 1993; Whittle et al., 2002; Bahl et al., 2004; Feld et al., 2008; Aminov, 2011; Lu et al., 2017). In our previous study, we designed an experimental setup for measurement of conjugation frequency in which we could separate conjugation rate from the power of selection by the antibiotics. We showed that the transfer frequency of the plasmid pTF2 in *Escherichia coli* MG1655 in an antibiotic free environment was increased significantly when the donor was pre-grown in broth containing cefotaxime (CTX) (Moller et al., 2017). However, the underlying mechanism remains to be determined.

TraF, an essential component of the *E. coli* T4SS, is responsible for the processing of pilus assembly, which is essential in the formation of mating apparatus and for conjugative plasmid transfer (Waters et al., 1992; Huang et al., 2019). Previous studies showed that modification of any region of *traF* abolished pilus synthesis, resulting in a loss of conjugative function (Lento et al., 2016). In the current study we used this gene fused to a *lacZ* reporter gene to identify genes not directly involved in the plasmid transfer mechanism and involved in the CTX-induced increased conjugative transfer of the ESBL encoding plasmid. We used a genetic screen, where random insertional mutagenesis was performed in *E. coli* MG1655/pTF2 with a *lacZ* reporter gene fused to the *traF* promoter on the plasmid. Our results identified six chromosomally encoded genes and one plasmid encoded gene involved in the CTX induced plasmid transfer mechanism. Such genes may be future targets to prevent antibiotic induced spread of resistance plasmids.

## Materials and Methods

### Bacterial Strains and Growth Conditions

Bacterial strains and plasmids used in this study are listed in Table 1. Luria-Bertani (LB) media was used in experiments for growth of bacteria. Bacterial strains were grown at 37°C except for strains containing the temperature sensitive plasmids, pKD46 and pCP20, which were grown at 30°C. When appropriate, media were supplemented with antibiotics (Sigma, Copenhagen, Denmark) including 20 mg/L gentamicin (Gem); 50 mg/L kanamycin (Kam); 25 mg/L chloramphenicol (Cap); 10 mg/L Trimethoprim (Tmp) and 2–512 mg/L cefotaxime (CTX). The β-galactosidase chromogenic indicator 5-bromo-4-chloro-3-indolyl β-D-galactopyranoside (X-gal) was used at a concentration of 80 mg/L.

**TABLE 1:** Bacterial strains and plasmids used in this study.

**Strains**	**Genotype**	**References**
MG1655/pTF2	*E. coli* MG1655 + *bla*_CTX–_*_M–_*_1_ containing IncI1 plasmid pTF2 (Amp^R^)	Kjeldsen et al., 2015
J53-2	*E. coli*, Rif^R^	Appelbaum et al., 1972
ATCC^®^ 25922	*E. coli* Reference strain	Ceri et al., 1999
GL100	MG1655/pTF2 Δ*traF*:*lacZ*, CTX^R^	This work
GL101	MG1655 *rfaH*:Tn5/pTF2 Δ*traF*:*lacZ*, CTX^R^, Tmp^R^	This work
GL102	MG1655 *yhiN*:Tn5/pTF2 Δ*traF*:*lacZ*, CTX^R^, Tmp^R^	This work
GL103	MG1655 *waaP*:Tn5/pTF2 Δ*traF*:*lacZ*, CTX^R^, Tmp^R^	This work
GL104	MG1655 *waaQ*:Tn5/pTF2 Δ*traF*:*lacZ*, CTX^R^, Tmp^R^	This work
GL105	MG1655 *gnd*:Tn5/pTF2 Δ*traF*:*lacZ*, CTX^R^, Tmp^R^	This work
GL106	MG1655 *pgl*:Tn5/pTF2 Δ*traF*:*lacZ*, CTX^R^, Tmp^R^	This work
GL107	MG1655/pTF2 Δ*traF*:*lacZ* IS*Ecp1*:Tn5, CTX^R^, Tmp^R^	This work
GL111	MG1655 Δ*rfaH*/pTF2, CTX^R^	This work
GL112	MG1655 Δ*yhiN*/pTF2, CTX^R^	This work
GL113	MG1655 Δ*waaP*/pTF2, CTX^R^	This work
GL114	MG1655 Δ*waaQ*/pTF2, CTX^R^	This work
GL115	MG1655 Δ*gnd*/pTF2, CTX^R^	This work
GL116	MG1655 Δ*pgl*/pTF2, CTX^R^	This work
GL117	MG1655/pTF2 ΔIS*Ecp1*, CTX^R^	This work
**Plasmids**		
pKD46	rep_pSC__101_^ts^ Gem^R^ P*_*araBAD*_*γβ *exo*	Doublet et al., 2008
pKD4	rep_R__6__K_ γAmp^R^ FRT Kam^R^ FRT	Datsenko and Wanner, 2000
pCP20	rep_pSC__101_^ts^ Amp^R^ Cap^R^ *cI*857 λP_R_	Cherepanov and Wackernagel, 1995
pCE36	rep_R__6__K_ γ Kam^R^ FRT lacZY t_his_	Ellermeier et al., 2002

### Antimicrobial Susceptibility Testing

The minimal inhibitory concentrations (MIC) of CTX was determined using the broth microdilution methods using 0–512 mg/L by 2-fold dilution increases, and using the control strain *E. coli* ATCC^®^ 25922 following the CLSI guidelines M100-S25 as previous described (Wayne and Clinical and Laboratory Standard Institute [CLSI], 2015).

### Construction of *lacZ* Reporter Fusions

A LacZ reporter fusion MG1655/pTF2 Δ*traF*:*lacZ* was created using the λ Red recombination method as previously described (Datsenko and Wanner, 2000; Ellermeier et al., 2002). Using the primers traF-F and traF-R, a PCR fragment of the kanamycin cassette (Kam^R^) from plasmid pKD4 was amplified using Phusion^TM^ Hot Start II DNA polymerase (ThermoFisher Scientific) and purified using the GeneJET PCR Purification Kit (ThermoFisher Scientific) and the fragment was introduced into MG1655/pTF2 harboring pKD46 by electroporation, to exchange the *traF* gene with the kanamycin cassette. The kanamycin resistant cassette was removed using plasmid pCP20, and the *lacZ* transcriptional fusion plasmid pCE36 was integrated into the tyrosine DNA recombinase (FLP) recombination target sequence at the deleted *traF* locus (Datsenko and Wanner, 2000; Ellermeier et al., 2002). Competent cells for electroporation was prepared by washing three times with ice-cold water and electroporation buffer (10% glycerol) at an optical density of 0.6 (OD_600_). 200 ng DNA was mixed with 50 μL of competent cells for electroporation at 25 μF, 200Ω, and 2.5 kV. Primer sequences can be seen in Supplementary Table S1.

### Transposon Mutant Library Generation

A transposon library was generated by electroporation of the EZ-Tn5^TM^ transposome into MG1655/pTF2 Δ*traF*:*lacZ*. EZ-Tn5^TM^ transposome complexes were formed between an EZ-Tn5^TM^ transposon (Epicenter) and EZ-Tn5^TM^ transposase (Epicenter), carrying a trimethoprim resistance marker to serve as a selection marker for transposon mutants. One μL of the EZ-Tn5^TM^ transposome was used for electroporation and bacteria were plated on LB agar plates containing 10 mg/L Tmp. Totally, 2 × 10^4^ transposon mutants were separated on the plates. The transposon library was stored as 30% glycerol stocks at −80°C.

### Screening of Transposon-Library

The transposon-library was plated on LB agar plates containing CTX (32 mg/L) and X-gal (80 mg/L) and screened for white/light blue colonies, corresponding to absence of CTX induced up-regulation of *traF*.

### β-Galactosidase Assay

β-galactosidase assays were carried out according to the method of Miller (1972). Overnight cultures were diluted 100 fold in LB broth and allowed to grow at 37°C. Two experiments were performed: (i) β -galactosidase activity at different optical density: MG1655/pTF2 Δ*traF*:*lacZ* cultures with and without CTX were grown at 37°C and 2 mL samples were collected at OD_600_ = 0.1, 0.3, 0.5, 0.7, 0.9, 1.1, 1.3, and 1.5; (ii) β -galactosidase assay of wild-type (MG1655/pTF2 Δ*traF*:*lacZ)* and transposon-mutants at OD_600_ = 0.5: MG1655/pTF2 Δ*traF*:*lacZ* and the seven transposon-mutants were grown with and without CTX to OD_600_ = 0.5 and 2 mL samples were collected. All samples were immediately cooled down and centrifuged for 3 min at 8000 rpm. Pellets were resuspended in 1 mL 100 mM Z-buffer (PH 7.0), permeabilized by adding 25 μL 0.1% (W/V) SDS and 50 μL chloroform and mixed by vortexing and incubated 5 min at room temperature. β-galactosidase assay was performed using 200 μL ONPG (4 mg/mL) (o-nitrophenyl-β-D-galactopyranoside) in Z-buffer. Samples were incubated at room temperature and when color change was observed, the reactions were terminated by the addition of 500 μL of 1 M Na_2_CO_3_. OD_420_ and OD_550_ was measured, and activity calculated in Miller-units = 1000 × (OD_420_-(1.75 × OD_550_))/(T × V × OD_600_). Data correspond to three independent assays conducted in duplicate, and all values are the mean ± S.D.

### Transposon Site Identification

Genomic DNAs were isolated from mutants using the MasterPure^TM^ complete DNA purification Kit (Epicenter) according to the instructions of the supplier. The identification of the transposon insertion site was done by whole genomic sequencing in an Illumina MiSeq (Illumina, Inc., San Diego, CA, United States) at a 300-bp paired-end-read format. Sequencing reads were *de novo* assembled using the SPAdes v.3.5.0 (Bankevich et al., 2012). Transposon insertions sites were identified using BLAST in CLC Main Workbench 8.0.0 (CLC bio, Denmark), and the locations of the transposon inserts were determined by a blastn comparison with the sequence of *E. coli* K-12 MG1655 (accession number U00096.3) (Altschul et al., 1990).

### Targeted Deletion Mutagenesis

Site specific gene deletion in MG1655/pTF2 was done by insertion of kanamycin cassettes by the Lambda red recombinase system (Doublet et al., 2008). Insertions were confirmed by PCR. The kanamycin cassette was then removed from the seven Kam^R^ mutants using pCP20 as previously described (Doublet et al., 2008). Primers used for generating and confirming mutations are listed in Supplementary Table S1.

### RNA Extraction and RT-qPCR

Single colonies of MG1655/pTF2 and the seven deletion mutants were grown overnight in LB media at 37°C. The cultures were diluted 1000 fold and grown with and without 1/2 MIC of CTX to OD_600_ = 0.5. A FastPrep cell disrupter system (Qbiogene, Illkirch, France) and RNeasy Mini Kit (Qiagen, Sollentuna, Sweden) was used to extract total RNA. RNA quantity was determined by NanoDrop 1000 spectrophotometer (Thermo Scientific, Hvidovre, Denmark). Genomic DNA was removed by TURBOTM DNase kit (2 U/μL) (Ambion, Life Technologies, Nearum, Denmark). Purified RNA was reverse-transcribed into cDNA using the High Capacity cDNA Reverse Transcription Kit (Life Technologies, Naerum, Denmark). RT-qPCR was performed using FastStart Essential DNA Green Master (Roche, Hvidovre, Denmark) and a LightCycler 96 (Roche, Hvidovre, Denmark) as described by Pfaffl (2001). *gapA* and *nusG*, which have previously been validated, were used as reference genes (Kjeldsen et al., 2015). RT-qPCR was performed twice on separate biological samples and the results were calculated by the 2^–ΔΔCt^ method. Primer sequences can be seen in Supplementary Table S1.

### Growth Experiment

Growth of MG1655/pTF2 and deletion mutants was evaluated without and with 1/2 MIC CTX. Growth curves were obtained in biological triplicate using the automated microbiology growth curve analysis system Bioscreen C^TM^ (Oy Growth Curves Ab Ltd, Finland). A final volume of 200 μL LB broth was inoculated with cells from overnight cultures to a final cell density of 5 × 10^5^ cfu/mL, using a Sensititre^TM^ Nephelometer (Thermo Scientific^TM^, Roskilde, Denmark) with a 0.5 McFarland turbidity standard. The OD_600_ was measured every 15 min with continuous shaking for 24 h at 37°C. OD values of blank samples were subtracted from sample OD values at the respective time points before analyzing the data.

### Conjugation Experiments

MG1655/pTF2 and deletion mutants were used as donors and *E. coli* J53-2 as recipient strain in the conjugation experiments aiming to determine the effect of targeted genes deletion on conjugational transfer rate. MG1655/pTF2 and its mutants were grown in LB media without and with 1/2 MIC of CTX to exponential phase (OD_600_ = 0.5). Antibiotics were removed by a washing steps and conjugation was performed by mixing donor and recipient strain in a 1:1 ratio on filters (0.22 μM, Millipore, Copenhagen, Denmark) on LB agar plates at 37°C for 30 and 60 min as previously described (Moller et al., 2017). The bacterial material was washed from the filters using isotonic NaCl and plated on LB agar plates containing 2 mg/L CTX (for quantifying donor + transconjugants) or 50 mg/L rifampicin and 2 mg/L CTX (quantifying transconjugants only) and incubated overnight at 37°C. The conjugation frequency was calculated as transconjugants divided by number of donors. The conjugation experiments were performed in three biological duplicates with three technical replicates each.

### Statistical Analyses

Statistical analysis was performed using the GraphPad Prism (GraphPad Software) version 7.03. Comparisons of gene expression and conjugation frequencies with and without antibiotics were performed by student’s *t*-test with Welch’s correction. A *P*-value of ≤0.05 was considered statistically significant.

## Results

### Identifying Genes Involved in CTX-Induced *traF* Expression

Our previous work have reported that the transfer genes and proteins involved in conjugation of the *bla*_CTX–M–__1_ plasmid pTF2 were significantly up-regulated when *E. coli* MG1655/pTF2 was treated with 1/2 MIC (126 mg/L) concentrations of CTX during growth (Moller et al., 2017). In order to identify genes involved in the mechanism by which CTX influence the Type IV secretion system (T4SS) and hence conjugation, MG1655/pTF2 Δ*traF*:*lacZ*, containing a LacZ reporter fused to the *traF* promoter, was constructed. Growing this strain with CTX on X-gal plates led to dark blue colonies, revealing high expression of *traF* during CTX exposure. A transposon library of MG1655/pTF2 Δ*traF*:*lacZ* with random Tmp-resistant Tn5 transposon insertions was screened on CTX and X-gal for lighter blue colonies, to identify genes involved in the CTX induced *traF* induction. A total of 14 light blue colonies were isolated.

In order to identify the transposon insert site, whole genome sequencing was performed on these 14 light blue mutants. Totally eight different insertion sites were identified, six of them located on the chromosome [*rfaH* (one isolate), *yhiN* (one isolate), *waaP* (six isolates in two different positions), *waaQ* (two identical isolates), *gnd* (one isolate), and *pgl* (two identical isolates)] and one on the plasmid [IS*Ecp1* (one isolate)] (Table 2 and Supplementary Figure S1).

**TABLE 2:** CTX responsive genes and their products.

**Gene**	**Product**	**Transposon insertion site^∗^**	**Gene access no.**
*rfaH*	Transcription antiterminator	82/83	M94889.1
*yhiN*	Putative FAD/NAD(P)-binding oxidoreductase	42/43	NC_000913.3
*waaP*	LPS core heptose (I) kinase	434/435 683/684	NC_000913.3
*waaQ*	LPS core heptosyltransferase III	607/608	NC_000913.3
*gnd*	6-phosphogluconate dehydrogenase	757/758	NC_000913.3
*pgl*	6-phosphogluconolactonase	850/851	NC_000913.3
IS*Ecp1*	IS*Ecp1* transposase	973/974	KJ563250

*^∗^Numbers refer to the base-position of the gene, between which the transposon has inserted.*

**FIGURE 1:**
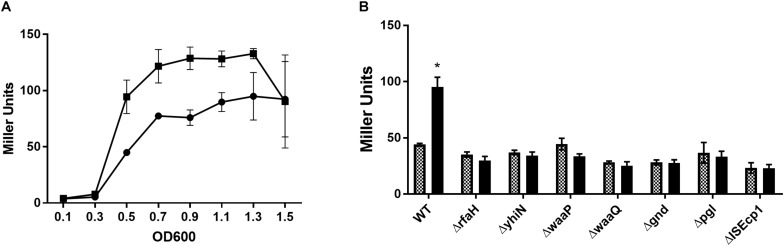
β-galactosidase activity increases in MG1655/pTF2 Δ*traF*:*lacZ* during CTX exposure, but not in the transposon-mutants. **(A)** LacZ expression of MG1655/pTF2 Δ*traF*:*lacZ* with (square) and without (Circle) CTX during growth. **(B)** LacZ expression of MG1655/pTF2 Δ*traF*:*lacZ* (WT) and the seven transposon-mutants without (squared bars) and with 1/2 MIC CTX (black bars) exposure at OD_600_ = 0.5. All experiments were carried out in triplicate, and each value is presented as the average plus standard deviation. The star indicates statistical significance between antibiotic treated and untreated at level: ^∗^*P* ≤ 0.05.

To confirm the reduced expression from the *traF* promoter, a β-galactosidase assay was performed, with and without CTX, using MG1655/pTF2 Δ*traF*:*lacZ* and the 7 transposon mutants. Measuring the changes in expression during growth, showed that the β-galactosidase level in MG1655/pTF2 Δ*traF*:*lacZ* was increased significantly from OD_600_ = 0.5 by CTX treatment (Figure 1A). Measuring the LacZ expression from MG1655/pTF2 Δ*traF*:*lacZ* and the seven transposon-mutants at OD_600_ = 0.5 revealed that CTX did not induce the *traF* promoter in the mutants, confirming the results from the transposon screen (Figure 1B).

**FIGURE 2:**
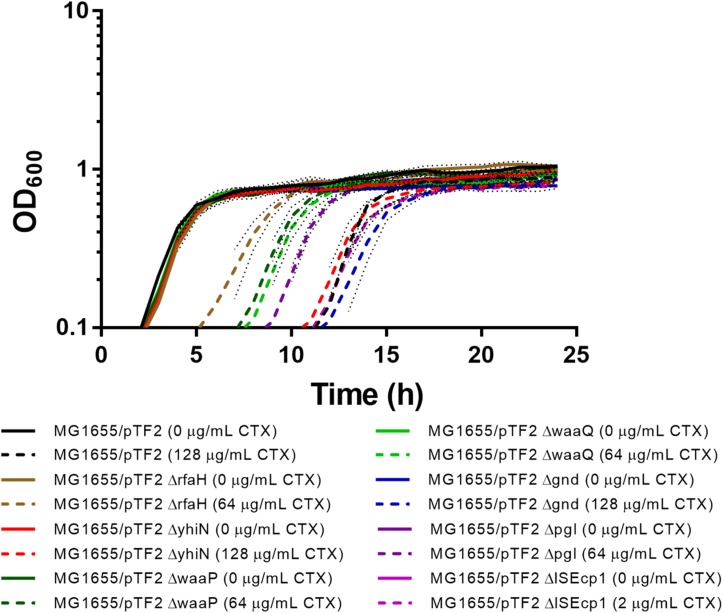
Growth phenotypes of MG1655/pTF2 and its mutants with and without CTX. All strains were grown without and with CTX (1/2 MIC of the corresponding strain). Three independent replicates were performed and the data shown represent the mean and dots represent standard deviations.

Individual deletions of the seven identified genes were constructed in MG1655/pTF2. In order to investigate whether the deletions had an impact on CTX resistance and on bacterial growth, the MIC of CTX for the mutants was determined and the growth pattern of the strains with and without 1/2 MIC CTX was investigated. The MIC of CTX for Δ*yhiN* and Δ*gnd* corresponded to the MIC of the wild-type (MG1655/pTF2), while MIC for Δ*rfaH*, Δ*waaP*, Δ*waaQ*, and Δ*pgl* decreased two fold from 256 mg/L to 128 mg/L and ΔIS*Ecp1* decreased seven fold from 256 mg/L to 4 mg/L. Without CTX exposure, the strains showed a similar growth pattern, however, when exposed to CTX (1/2 MIC of the corresponding strain), the Δ*rfaH*, Δ*waaP*, Δ*waaQ*, and Δ*pgl* showed decreased length of lag phase compared to MG1655/pTF2 (Figure 2). However, since all expression and conjugation experiments were performed with bacteria grown to the same OD, the variations in lag-phase were not expected to influence the obtained results.

**FIGURE 3:**
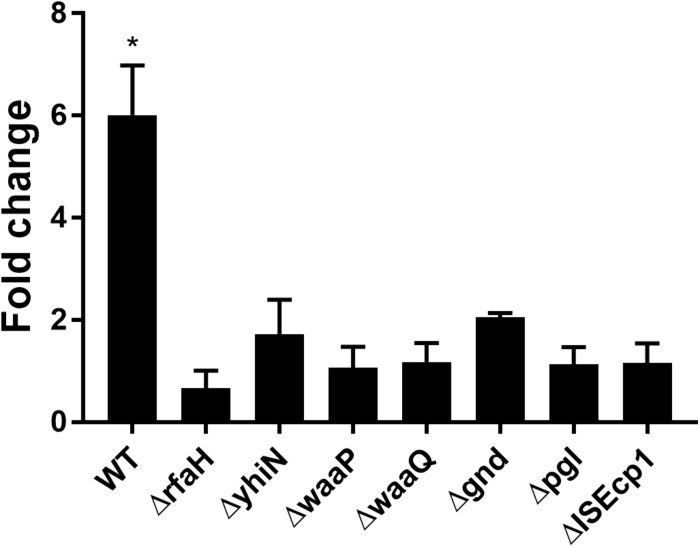
CTX induced expression of the *traF* gene in WT (MG1655/pTF2) and deletion mutants. Strains were grown with 1/2 MIC CTX or without. Data are presented as fold change in CTX induced expression relative to expression levels without CTX. Three independent replicates including two technical replicates each were performed. The data shown represents the mean and the error bars represent standard deviations. The expression data was normalized to *gapA* and *nusG*. The stars indicate statistical significance between the two conditions at level: ^∗^*P* ≤ 0.05.

To confirm the importance of the genes in CTX induced *traF* regulation, we used RT-qPCR analysis, and found a significant up-regulation of *traF* (6.1-fold, *t*-test, *P* = 0.01) when the wild-type strain (MG1655/pTF2) was treated with CTX. In contrast, and in support of the β-galactosidase assay results, none of the deletion mutants showed significant CTX induced *traF* up-regulation (Figure 3).

### CTX-Induced *bla*_CTX–M–__1_ Expression

**FIGURE 4:**
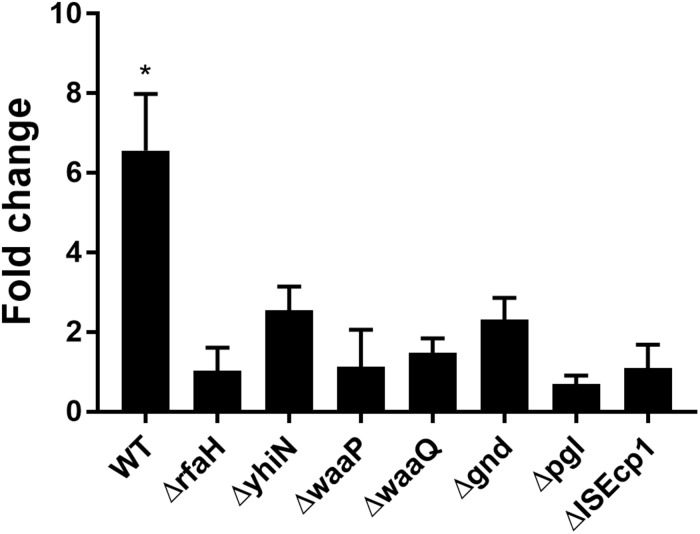
CTX induced expression of *bla*_CTX–M–1_ in WT (MG1655/pTF2) and deletion mutants. Strains were grown with 1/2MIC CTX or without. Data are presented as fold change in CTX induced expression relative to expression levels without CTX. Two independent replicates including two technical replicates each were performed. The data shown represents the mean and the error bars represent standard deviations. The expression data was normalized to two validated reference genes, *gapA* and *nusG*. The stars indicate statistical significance at level: ^∗^*P* ≤ 0.05.

We have previously reported that the induction of transfer gene-expression and increased conjugation frequency by treatment with CTX is dependent on the presence of the antibiotic resistance gene *bla*_CTX–M–__1_ (Moller et al., 2017). To investigate whether the identified seven genes also work in a CTX-M-1 dependent manner to regulate the CTX-induced increased conjugation, we performed RT-qPCR. We found that the *bla*_CTX–M–__1_ gene expression was significantly up-regulated (6.56- fold, *t*-test, *P* = 0.03) in the wild-type strain during CTX treatment, whereas CTX exposure had limited, non-significant, effect on *bla*_CTX–M–__1_ expression in the seven knock-out mutants (Figure 4) and the seven isolated transposon-mutants (data not shown). Expression of *bla*_CTX–M–__1_ was not significantly different between the mutants and the wild-type, when the strains were not exposed to CTX (Supplementary Figure S2).

### CTX-Induced Expression of Selected Gene in Wild-Type and Δ*rfaH* Mutant

**FIGURE 5:**
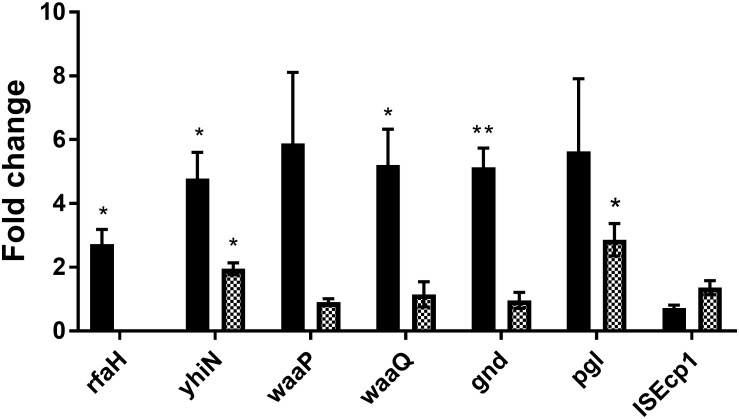
CTX induces expression of six genes involved in antibiotic induced *traF* expression in MG1655/pTF2. MG1655/pTF2 (Black bars) and the Δ*rfaH* (squared bars) were grown with 1/2 MIC CTX or without. Data are presented as fold change in CTX induced expression relative to expression levels without CTX. Three independent replicates including two technical replicates each were performed. The data shown represents the mean and the error bars represent standard deviations. The expression data was normalized to *gapA* and *nusG*. The stars indicate statistical significance at different levels: ^∗^*P* ≤ 0.05, ^∗∗^*P* ≤ 0.01.

In order to investigate whether the genes identified by transposon mutagenesis were regulating *tra* gene expression independent of CTX or not, RT-qPCR was performed to investigate the expression of the seven genes in the wild-type strain, comparing expression between CTX treated and untreated wild-type. Significant up-regulation of *rfaH* (2.7- fold, *t*-test, *P* = 0.03), *yhiN* (4.8- fold, *t*-test, *P* = 0.02), *waaQ* (5.2- fold, *t*-test, *P* = 0.03) and *gnd* (5.1- fold, *t*-test, *P* = 0.006) was observed, when the wild-type was treated with 1/2 MIC of CTX during growth. For the *waaP* and *pgl* genes, a non-significant, 5.9- and 5.6- fold (*t*-test, *P* > 0.05) increase in gene expression was observed. The data showed that CTX do up-regulate the expression of these genes, except the IS*Ecp1* (Figure 5, black bars). In addition, we investigated the expression level of the individual genes in the *rfaH* mutant background, to evaluate a possible regulatory link between RfaH and the remaining genes. Only *yhiN* (2.0- fold, *t*-test, *P* = 0.01) and *pgl* (2.9- fold, *t*-test, *P* = 0.03) showed significant up-regulation when Δ*rfaH* was treated with CTX during growth, however, not to the level observed in the wild-type (Figure 5, squared bars).

### CTX-Induced Conjugative Plasmid Transfer

In order to investigate whether the deletion of the seven genes would affect the number of CTX-induced conjugation events, conjugation experiments was performed with the wild-type (MG1655/pTF2) and the mutants, pre-grown with or without CTX. The data showed that the CTX treatment significantly increased plasmid transfer frequency in MG1655/pTF2 with 25.0- and 41.0- fold (*t*-test, *P* ≤ 0.05) after 30 and 60 min of cell contact, relative to conjugation without pre-growth in the presence of CTX (Table 3). In contrast, all of the mutants revealed a decreased CTX-induced plasmid transfer compared to the wild-type. We did not obtain any transconjugants for ΔIS*Ecp1* probably due to transfer frequencies below detection limit.

**TABLE 3:** Fold changes of CTX-induced (1/2 MIC) increased conjugation transfer frequency, relative to untreated strains, using MG1655/pTF2 (WT) and the mutants as donors and J53-2 as recipient.

**Donor**	**Conjugation time (min)**	**Conjugation transfer frequency (Control)^a^**	**Conjugation transfer frequency (CTX)^a^**	**Plasmid transfer fold difference^b^**
WT	30	3.90 × 10^–5^ ± 1.05 × 10^–5^	9.75 × 10^–4^ ± 1.46 × 10^–4^	25.00^∗∗^
	60	3.00 × 10^–4^ ± 0.49 × 10^–4^	1.23 × 10^–2^ ± 0.34 × 10^–2^	41.00^∗^
Δ*rfaH*	30	1.13 × 10^–5^ ± 0.32 × 10^–5^	0.87 × 10^–5^ ± 0.26 × 10^–5^	0.77
	60	1.14 × 10^–4^ ± 0.12 × 10^–4^	1.06 × 10^–4^ ± 0.39 × 10^–4^	0.93
Δ*yhiN*	30	2.48 × 10^–5^ ± 0.96 × 10^–5^	3.64 × 10^–4^ ± 0.39 × 10^–4^	14.68^∗∗^
	60	5.25 × 10^–4^ ± 1.24 × 10^–4^	2.86 × 10^–3^ ± 0.60 × 10^–3^	4.50^∗^
Δ*waaP*	30	2.33 × 10^–5^ ± 0.65 × 10^–5^	3.46 × 10^–4^ ± 0.72 × 10^–4^	14.85^∗^
	60	3.29 × 10^–4^ ± 0.79 × 10^–4^	3.09 × 10^–3^ ± 0.97 × 10^–3^	9.57
Δ*waaQ*	30	2.97 × 10^–5^ ± 1.04 × 10^–5^	3.23 × 10^–5^ ± 0.67 × 10^–5^	1.09
	60	2.73 × 10^–4^ ± 0.60 × 10^–4^	4.47 × 10^–3^ ± 1.44 × 10^–3^	16.37
Δ*gnd*	30	2.44 × 10^–5^ ± 0.64 × 10^–5^	3.16 × 10^–4^ ± 0.65 × 10^–4^	12.95^∗^
	60	3.59 × 10^–4^ ± 0.88 × 10^–4^	5.47 × 10^–3^ ± 0.4707 × 10^–3^	15.24^∗∗^
Δ*pgl*	30	1.16 × 10^–5^ ± 0.24 × 10^–5^	1.26 × 10^–4^ ± 0.24 × 10^–4^	10.86^∗^
	60	2.27 × 10^–4^ ± 0.48 × 10^–4^	2.31 × 10^–3^ ± 0.41 × 10^–3^	10.18^∗^

*^*a*^The results shown are from two biological replicates with three technical replicates each, the data shown represent the mean ± standard deviation. ^*b*^The stars indicate statistical significance at level: ^∗^*P* ≤ 0.05, ^∗∗^*P* ≤ 0.01.*

## Discussion

The contribution of antibiotics as a stimulating factor to the promotion of conjugation transfer has previously been investigated (Barr et al., 1986; Bahl et al., 2004; Feld et al., 2008; Lu et al., 2017; Moller et al., 2017). However, despite our current knowledge that antibiotics can increase conjugation frequency, it still remains unclear which mechanisms are involved in this phenomenon. We have previously obtained evidence that 1/2 MIC concentrations of CTX affects expression of the conjugation apparatus of plasmid pTF2, as *tra*-genes were significantly up-regulated at both the transcriptional and translational level (Moller et al., 2017). *traF* is one of several T4SS proteins involved in pilus assembly and essential for plasmid transfer (Frost et al., 1994). In the current study, a *traF*:*lacZ* reporter gene-fusion was constructed, to enable a screening for genes which affect the antibiotic induced up-regulation of *traF* expression. Random insertional mutagenesis mediated by Tn5 transposon was carried out in the reporter strain and in total seven genes were identified (*rfaH*, *yhiN*, *waaP* (*rfaP*), *waaQ* (*rfaQ*), *gnd*, and *pgl* and the pTF2 plasmid-encoded insertion sequence IS*Ecp1*) where knock-out by transposon insertion abolished the CTX induced up-regulation of *traF*.

RfaH is a transcriptional antiterminator, which activates operons encoding lipopolysaccharide (LPS) core components, pili, toxins, capsules and antibiotic biosynthesis in *Enterobacteriaceae* (Sanderson and Stocker, 1981; Bailey et al., 2000; Nandymazumdar and Artsimovitch, 2015). Furthermore, reduced plasmid transfer has been shown for a *Salmonella Typhimurium rfaH* mutant (Sanderson and Stocker, 1981). A possible explanation for CTX induced up-regulation of *traF* is therefore that CTX treatment increases the expression of *rfaH*, and this activates the expression of pilus encoding genes, such as *traF*. A previous study has shown RfaH binding to an ops element in the promoter region of the *tra* operon on the F plasmid (Bailey et al., 1997). Sequence analysis revealed an ops element in the *tra* promoter region on pTF2 (IncI1 plasmid), supporting direct RfaH regulation of *traF* expression. Furthermore, we showed that CTX induce *rfaH* and *traF* expression, however, the latter not in a *rfaH* knock out background, and thus that the CTX induction of the *traF* expression is dependent on RfaH. The *waaP* and *waaQ* genes, which are regulated by RfaH, play important roles in LPS biosynthesis and the formation of a stable outer membrane (Yethon et al., 1998; Belogurov et al., 2009). Mutations in the *waa* locus can significantly alter outer membrane permeability and hypersensitivity to detergents and hydrophobic antibiotics (Schnaitman and Klena, 1993). *gnd* and *pgl* are two enzymes of the oxidative branch of the hexose monophosphate shunt in the pathway of glucose metabolism (Kupor and Fraenkel, 1969). It is unknown how *gnd* and *pgl* regulate *tra* expression, but *gnd* is adjacent to a RfaH-regulated transcription unit, suggesting RfaH affects expression of *gnd* (Belogurov et al., 2009). The *yhiN* gene is a putative FAD/NAD(P) binding oxidoreductase with unknown function. It is part of the RpoS regulon, indicating an importance in the stress response (Vijayakumar et al., 2004). Previously published results have shown that high levels of RpoS affect conjugative transfer in *Pseudomonas knackmussii* (Miyazaki et al., 2012). The *bla*_CTX–M_ genes are often associated with IS*Ecp1*-like elements (Karim et al., 2001; Baraniak et al., 2002; Chanawong et al., 2002; Poirel et al., 2003). These elements contain putative -35 and -10 promoter regions within the 3′ end of IS*Ecp1* affecting the expression level of the *bla*_CTX–M_ gene (Karim et al., 2001; Poirel et al., 2003).

Deletions of the seven genes were performed in MG1655/pTF2 to evaluate their involvement in CTX induced increased plasmid transfer. For the wild-type MG1655/pTF2 we observed a significant CTX induction of expression of *traF* and *bla*_CTX–M–__1_, and a significant induction of plasmid transfer. In contrast, we saw that the mutants were hampered in CTX-induced *traF* and *bla*_CTX–M–__1_ expression and plasmid transfer. Results confirmed that increased *bla*_CTX–M–__1_ gene expression is necessary for increased conjugation transfer frequency. We have previously shown that exposing a *bla*_CTX–M–__1_ mutant of MG1655/pTF2 to CTX did not lead to induced *tra* gene expression and plasmid transfer (Moller et al., 2017). Furthermore, when exposed to CTX, the ΔIS*Ecp1* mutant expressed very low levels of *bla*_CTX–M–__1_, as seen from the MIC, and was unable to induce *traF* expression. IS*Ecp1*-like elements have been shown to contain promoter sequences for high level expression of *bla*_CTX–M_ β-lactamase genes (Poirel et al., 2003); probably explaining why we observed limited CTX induced expression of *bla*_CTX–M–__1_. The IS*Ecp1* most likely is not part of the CTX induced conjugation pathway, instead the deletion simply affects *bla*_CTX–M–__1_ expression, supported by the severely reduced MIC, and the lack of CTX induced *traF* and *bla*_CTX–M–__1_ expression.

Our results showed that the deletion of the genes resulted in reduced CTX induction of plasmid transfer compared to the wild-type (MG1655/pTF2), confirming that the *rfaH*, *yhiN*, *waaP*, *waaQ*, *gnd* and *pgl*, genes are involved in the CTX induced increased pTF2 plasmid conjugative transfer.

In order to investigate how the seven identified genes contribute to CTX induced *traF* expression, we measured whether the genes themselves were regulated by CTX. We found that the expression of the *rfaH*, *yhiN*, *waaQ*, and *gnd* genes in the wild-type indeed were up-regulated significantly in the presence of CTX, and the expression of *waaP* and *pgl* was also up-regulated, although not significant. Only expression of IS*Ecp1* was not affected by CTX. This CTX-induced up-regulation of *waaP*, *waaQ*, and *gnd* disappeared in an *rfaH* mutant background, and the CTX induced expression of *yhiN* and *pgl* was decreased in the Δ*rfaH* strain compared to the expression levels in the wild-type background. These results support that RfaH is central not only in the regulation of these five genes, but also in the CTX induction of conjugation. Thus our current model is that CTX induce *bla*_CTX–M–__1_ expression as well as *rfaH* expression. This affects *tra* expression directly as well as through changed expression of the *waaP*, *waaQ*, *gnd, yhiN*, and *pgl* genes, which affect *bla*_CTX–M–__1_ expression and hence *traF* expression.

## Conclusion

In conclusion, six genes involved in CTX induced increased conjugation have been identified. Further experiments are needed to uncover the role of these genes in the pathways by which bacteria sense CTX and signals induced *tra* expression.

## Data Availability Statement

The raw data supporting the conclusions of this manuscript will be made available by the authors, without undue reservation, to any qualified researcher.

## Author Contributions

GL has performed all the experiments. LT, JO, and GL have participated in the design of the study and have participated in the article preparation. All authors have approved the final article.

## Conflict of Interest

The authors declare that the research was conducted in the absence of any commercial or financial relationships that could be construed as a potential conflict of interest.
