# Supplementary material for: Identification of Genes Essential for Antibiotic-Induced Up-Regulation of Plasmid-Transfer-Genes in Cephalosporin Resistant Escherichia coli
Source: Front Microbiol. 2019 Sep 24;10:2203. doi: 10.3389/fmicb.2019.02203 (PMC6768964; doi:10.3389/fmicb.2019.02203)
Supplement: Supplementary file 1 [file Table_1.docx]

Supplementary Material

**Supplementary Table 1**. Sequences of oligonucleotides used for RT-qPCR, Lambda Red mediated mutagenesis and PCR verifications.

| Primer | Sequence | Application |
| --- | --- | --- |
| traF-F | 5’-ATAATACCCCCGGGTTTAGTGCCGGGGGCGGGGACTGTAAGTGTAGGCTGGAGCTGCTTC-3’ | Knockout |
| traF-R | 5’-TAGCGTTGTTTTGTCTAAATTCTGATAAACAGGAAATTAACATATGAATATCCTCCTTAG-3’ |  |
| traF-check-F | 5’- CCAGGGTGATATCGGGAAGC-3’ | Proof of knockout |
| traF-check-R | 5’- TAATACAGGACCCGGCCAGA-3’ |  |
| rfaH-F | 5’-TTGCTATCCTTGCGCCCCGATTAAACGGATAAGAGTCATT GTGTAGGCTGGAGCTGCTTC-3’ | Knockout |
| rfaH-R | 5’-GGATGCTAATGTCAAAACACTGTTTGGGATTGCGTTTTAGCATATGAATATCCTCCTTAG-3’ |  |
| rfaH-check-F | 5’- TTTTCGAACAACAGCGCAGG-3’ | Proof of knockout |
| rfaH-check-R | 5’- GCGTGGCGCATGGAATTTAT-3’ |  |
| rfaH-qPCR-F | 5’-GTCAGTGAGCCATTGTTC-3’ | RT-qPCR |
| rfaH-qPCR-R | 5’-CGTTGATAGTCGTGGTATG-3’ |  |
| yhiN-F | 5’-CTTTACAATTCACGCCCGTTTTTTCTAAGAGGAGCGCAAC GTGTAGGCTGGAGCTGCTTC-3’ | Knockout |
| yhiN-R | 5’-TTTAATGCGACTCTAATAATTTTCATCTTTAGGAAATAGGCATATGAATATCCTCCTTAG-3’ |  |
| yhiN-check-F | 5’-TGAGTGCATCCACCACTGAC-3’ | Proof of knockout |
| yhiN-check-R | 5’-GACCATTTGCGTAACGCTGG-3’ |  |
| yhiN-qPCR-F | 5’-TGAGCCAGAATCCGCATT-3’ | RT-qPCR |
| yhiN-qPCR-R | 5’-GCCGTGTTTATTGACCAGAT-3’ |  |
| waaP-F | 5’-AAAAGCCGCGGATATCATTACAGGTGGTTTAGATGGTTGA GTGTAGGCTGGAGCTGCTTC-3’ | Knockout |
| waaP-R | 5’-TCATAATAAAGTTAGTTCCAGTACATACTAATAAATATTTCATATGAATATCCTCCTTAG -3’ |  |
| waaP-check-F | 5’-TGAAGCGCTGGCAGAAAAAC-3’ | Proof of knockout |
| waaP-check-R | 5’-GACGCCCTTTATCACGACCA-3’ |  |
| waaP-qPCR-F | 5’-GGCGTTGATACAATGAAG-3’ | RT-qPCR |
| waaP-qPCR-R | 5’-GGCGTTGATACAATGAAG-3’ |  |
| waaQ-F | 5’-GCTAGTGGAAAAGCCATTTCGAAAAATCCTGGTCATAAAGGTGTAGGCTGGAGCTGCTTC-3’ | Knockout |
| waaQ-R | 5’- CAAATGGAAAATATTTATATAAACAAAACGCCACGATCATCATATGAATATCCTCCTTAG-3’ |  |
| waaQ-check-F | 5’-ACGGCAGATAAACGTGCTGA-3’ | Proof of knockout |
| waaQ-check-R | 5’-TCTGCGGGATGCTCTTTGAG-3’ |  |
| waaQ-qPCR-F | 5’-CTTGAGCCATTAGGTATTAC-3’ | RT-qPCR |
| waaQ-qPCR-R | 5’-CTTGAGCCATTAGGTATTAC-3’ |  |
| gnd-F | 5’-GAGCATTCAGCGCGGTGATCACACCTGACAGGAGTATGTAGTGTAGGCTGGAGCTGCTTC-3’ | Knockout |
| gnd-R | 5’-ATATACGCCGGGCCTCAATTTTATTGTTGGTTAAATCAGACATATGAATATCCTCCTTAG-3’ |  |
| gnd-check-F | 5’- TTGACCTGTGCTTGAGGCTT-3’ | Proof of knockout |
| gnd-check-R | 5’- TGAACGCTCACCGATGACAA-3’ |  |
| gnd-qPCR-F | 5’-GGATGCTGCTATTGATTC-3’ | RT-qPCR |
| gnd-qPCR-R | 5’-TCACGATTACGACGAATA-3’ |  |
| pgl-F | 5’-TTAGCTGTTACAGTCAGTTGCTAAATGCAAAGGAGCATTCGTGTAGGCTGGAGCTGCTTC-3’ | Knockout |
| pgl-R | 5’-TACACCGGCGCAGGAGAGACTGCGCCGGGTAAATCAGCGGCATATGAATATCCTCCTTAG-3’ |  |
| pgl-check-F | 5’- CCAGCACGGTTTTTGCATGA-3’ | Proof of knockout |
| pgl-check-R | 5’- TCCATTGGCCAAAAAGCAGC-3’ |  |
| pgl-qPCR-F | 5’-ATATGCGTATTGCGTCAA-3’ | RT-qPCR |
| pgl-qPCR-R | 5’-TGGACACATTCGATATTACC-3’ |  |
| ISEcp1-F | 5’- AAAATAATAGTAAGAAAAGTAGTAAAAAGGGGTTCTAATT GTGTAGGCTGGAGCTGCTTC-3’ | Knockout |
| ISEcp1-R | 5’- GAATAGTCTGTCAAAACGCCTTTTTCTATTTATAGTCTAACATATGAATATCCTCCTTAG-3’ |  |
| ISEcp1-check-F | 5’-CGCAGGCTGTTTTCAACGAA-3’ | Proof of knockout |
| ISEcp1-check-R | 5’-AGCGGCACACTTCCTAACAA-3’ |  |
| ISEcp1-qPCR-F | 5’-CATTGGCATTGATAAGTTAG-3’ | RT-qPCR |
| ISEcp1-qPCR-R | 5’-GGTTGTCTTGAAGTTGAA-3’ |  |
| CTX-M-1-F | 5’-GACTATGGCACCACCAACG-3’ | RT-qPCR |
| CTX-M-1-R | 5’-GCTTTCTGCCTTAGGTTGAGG-3’ |  |
| K2 | 5’-CGGTGCCCTGAATGAACTGC-3’ | Proof of Kam insertion |
| Kt | 5’-CGGCCACAGTCGATGAATCC-3’ |  |
| gapA-F | 5’-ACTGACTGGTATGGCGTTCC-3’ | RT-qPCR Reference |
| gapA-R | 5’-GTTGCAGCTTTTTCCAGACG-3’ |  |
| nusG-F | 5’-GTCCGTTCGCAGACTTTAAC-3’ | RT-qPCR Reference |
| nusG-R | 5’-GCTTTCTCAACCTGACTGAAG-3’ |  |

**Supplementary Figure 1**. The location of transposon insertion sites within each gene. Numbers refer to the base-position of the gene, between which the transposon has inserted.


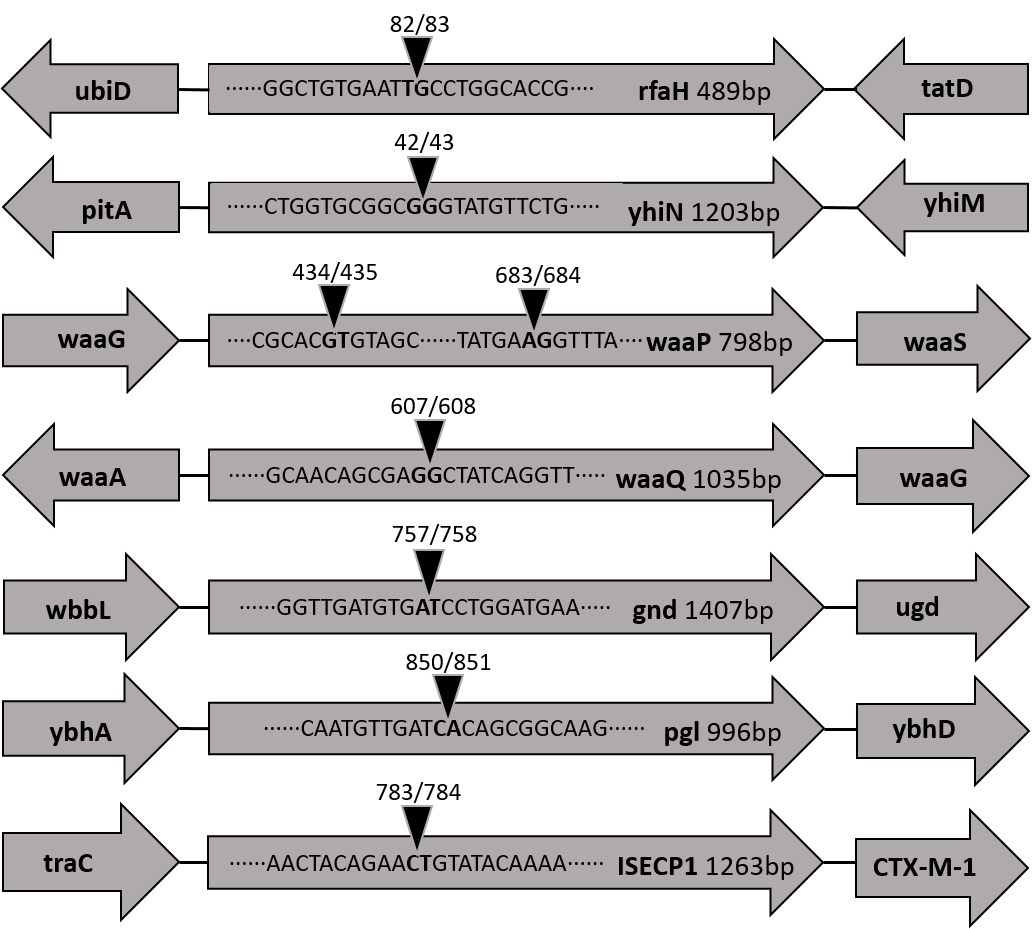


**
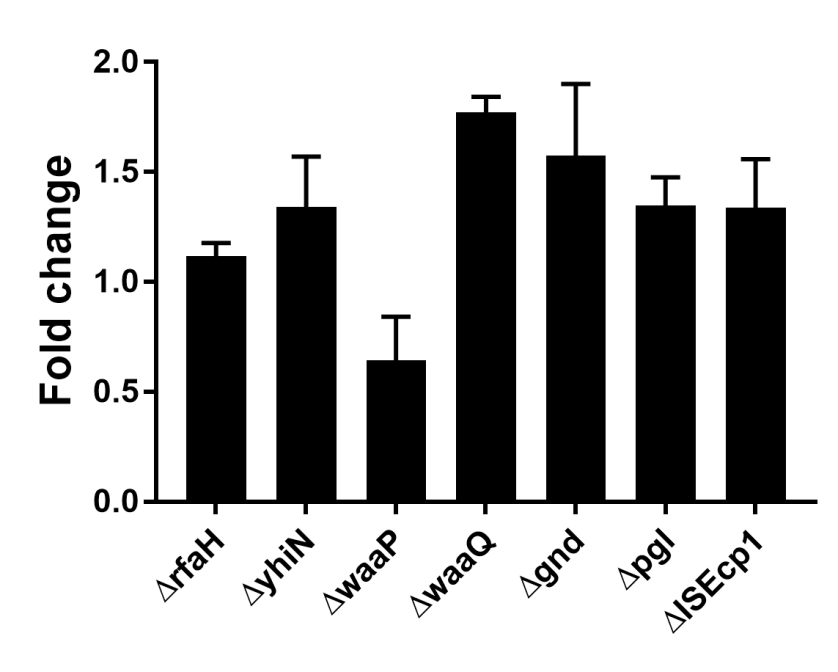
**

**Supplementary Figure 2**. Fold change of *bla*_CTX-M-1_ expression in the deletion mutants without CTX exposure relative to the WT expression level. Two independent replications including two technical replicates each were performed. The data shown represents the mean and the error bars represent standard deviations. The expression data was normalized to two validated reference genes, *gapA* and *nusG*.
